# A Curious Fact

**Published:** 1881-12

**Authors:** 


A FOREIGN scientific journal remarks as a curious physiological fact, and an important one, too, if true, that although open-air life is so favorable to health it has the apparent effect of stunting the growth in early youth. Thus, while the children of well-to-do parents, carefully housed and tended, are found to be taller for their age than the children of the poor, they are not so strong in after years. The laborer's children, who play in the lonely country roads and fields all day, whose parents lock their doors when leaving for work in the morning, so that their offspring shall not gain entrance and do mischief, are almost invariably short for their age ; and the children of working farmers exhibit the same peculiarity. But after the years of sixteen or eighteen the latter shoot up, and become great, hulking, broad fellows, possessed of immense strength. The conclusion, if correct, is clear—that indoor life forces growth at the wrong period, and that the natural parental pride in a big, bouncing child is altogether misplaced. A small, wiry, boy would seem to have a great advantage in the long run over his big and flabby rival.

